# Supplementary material for: Automated cell-type classification in intact tissues by single-cell molecular profiling
Source: eLife. 2018 Jan 10;7:e30510. doi: 10.7554/eLife.30510 (PMC5802843; doi:10.7554/eLife.30510)
Supplement: Supplementary file 1. [file elife-30510-supp1.docx]

**Supplementary File 1**

Connector circles

| CC06-DP4 | TTCCTGACCTAACAAAACATGCGTCTATTTAGTGGAGCCACATAATTATAACCTGGCTAT |
| --- | --- |
| CC03-X1 | TCGAACGAATAACAAAGGGAGACTGATTACACGATAACAACAATACACACAATACGTCGG |
| CC02-Y1 | TAGACGTACGAGATTAAAACATAGAGGCACTTGAGCTAGAATCATATAACAGGTTGTTCT |
| CC04-X4 | TCGCCTACTAAACAAAGAACCTCCATTAACATCGCAACAACAATACACACGCACTTATGT |
| CC05-DP5 | TGTTAGCGCTAACAAAATGCTGCTGCTGTACTACGAACAACAATACACATGTTACGACGT |
| CC07-Y7 | GACAATTACACGACTAAAACATCTCGGTGCTTGAACTGGTCTCAACTAACCTGAGGAATA |
| CC11X-X8 | TAACACGAACACTAAACAAGTCTA ACT AACGAT ACCATAAAGAAGAATAACCGAATCAAGAT |
| CC12X-X9mod | ACCATACTTGAACAAACCACACTCACATGGATCCACTCGTAACAACAATAACAGGATACATC |

Common Bridge

| CB2-31AC | TCAACTCGACGTATAACATAACGACGTAAGT |
| --- | --- |

Detection oligonucleotides

| AF488-Y1 | /5Alex488N/AGAGGCACTTGAGCTAGAAT |
| --- | --- |
| Cy5-DP5 | /5Cy5/AATGCTGCTGCTGTACTACGG |
| X1-Cy3 | GGGAGACTGATTACACGATA/3Cy3Sp/ |
| TEX615-X4 | /5TEX615/AGAACCTCCATTAACATCGC |
| Detection oligos with deoxy-U |  |
| uY7-A488 | /5Alex488N/CUCGGUGCUUGAACUGGUCU |
| uX8-Cy3 | GUCUAACUAACGAUACCAUA/3Cy3Sp/ |
| uX9-Texas red | /5Tex615/CUCACAUGGAUCCACUCGU |
| uDP4-Cy5 | /5Cy5/AUGCGUCUAUUUAGUGGAGCC |

| **Fluorphore** | **Compatible CC** | **H Probes (2X, 3X, 4X, 5X, 6X,7X,11X, 12X)** | **Design Templates for H Probes (2X, 3X, 4X, 5X, 6X, 7X, 11X, 12X)** |
| --- | --- | --- | --- |
| DP5- Cy5 | CC05-DP5 + CB2-31AC | HL5X-Gene-Position | TAGCGCTAACAACTTACGTCGTTATG:LLLLL |
|  |  | HR5X-Gene-Position | RRRRR:TTATACGTCGAGTTGAACGTCGTAACA |
| Y1-A488 | CC02-Y1 + CB2-31AC | HL2X-Gene-Position | TCGTACGTCTAACTTACGTCGTTATG:LLLLL |
|  |  | HR2X-Gene-Position | RRRRR:TTATACGTCGAGTTGAAGAACAACCTG |
| X1-Cy3 | CC03-X1 + CB2-31 AC | HL3X-Gene-Position | TATTCGTTCGAACTTACGTCGTTATG:LLLLL |
|  |  | HR3X-Gene-Position | RRRRR:TTATACGTCGAGTTGACCGACGTATTG |
| X4- Texas Red | CC04-X4 + CB2-31AC | HL4X-Gene-Position | TTAGTAGGCGAACTTACGTCGTTATG:LLLLL |
|  |  | HR4X-Gene-Position | RRRRR:TTATACGTCGAGTTGAACATAAGTGCG |
| uDP4-Cy5 | CC06-DP4 + CB2-31AC | HL6X-Gene-Position | TAGGTCAGGAAACTTACGTCGTTATG:LLLLL |
|  |  | HR6X-Gene-Position | RRRRR:TTATACGTCGAGTTGAATAGCCAGGTT |
| uY7-A488 | CC07-Y7 + CB2-31AC | HL7X-Gene-Position | GTGTAATTGTCACTTACGTCGTTATG:LLLLL |
|  |  | HR7X-Gene-Position | RRRRR:TTATACGTCGAGTTGATATTCCTCAGG |
| uX8- Cy3 | CC11X-X8+CB2-31AC | HL11X-Gene-Position | TGTTCGTGTTAACTTACGTCGTTATG:LLLLLLL |
|  |  | HR11X-Gene-Position | RRRRRR:TTATACGTCGAGTTGAATCTTGATTCGGT |
| uX9-Texas Red | CC12X-X9mod +CB2-31AC | HL12X-Gene-Position | TCAAGTATGGTACTTACGTCGTTATG:LLLLL |
|  |  | HR12X-Gene-Position | RRRRR:TTATACGTCGAGTTGAGATGTATCCTG |

Mouse – PLISH probes

| mm-HL4X-Ager-1335 | TTAGTAGGCGAACTTACGTCGTTATGTCTGGTTGGAGAAGGAAGTG |
| --- | --- |
| mm-HR4X-Ager-1335 | AGCATGGATCATGTGGGCTTATACGTCGAGTTGAACATAAGTGCG |
| mm-HL4X-Ager-1280 | TTAGTAGGCGAACTTACGTCGTTATGTGCTCTAGGGCCATCACAC |
| mm-HR4X-Ager-1280 | GATGGAATGTGGGGGAGCTTATACGTCGAGTTGAACATAAGTGCG |
| mm-HL4X-Ager-1031 | TTAGTAGGCGAACTTACGTCGTTATGTCACCCACAGAGCCTTCAG |
| mm-HR4X-Ager-1031 | CTAGCGTACCCAGCCCAGACTTATACGTCGAGTTGAACATAAGTGCG |
| mm-HL4X-Ager-771 | TTAGTAGGCGAACTTACGTCGTTATGTATTCCACCTTCAGGCTCAA |
| mm-HR4X-Ager-771 | AGTCCCACCAGGAGCGACTTATACGTCGAGTTGAACATAAGTGCG |
| mm-HL4X-Ager-652 | TTAGTAGGCGAACTTACGTCGTTATGTGCAGGAGAAGGTAGGATGG |
| mm-HR4X-Ager-652 | GAAGGCCCAGGCTGAAACTTATACGTCGAGTTGAACATAAGTGCG |
| mm-HL4X-Ager-496 | TTAGTAGGCGAACTTACGTCGTTATGTAAGGGTCCCTGCAGGGTAG |
| mm-HR4X-Ager-496 | TTTCCCATCTAAGTGCCAGCTTATACGTCGAGTTGAACATAAGTGCG |
| mm-HL4X-Ager-436 | TTAGTAGGCGAACTTACGTCGTTATGTGGCTGTGAGTTCAGAGGC |
| mm-HR4X-Ager-436 | TCCCCACCTTATTAGGGACACTTATACGTCGAGTTGAACATAAGTGCG |
| mm-HL4X-Ager-350 | TTAGTAGGCGAACTTACGTCGTTATGTTCCCTCGCCTGTTAGTTG |
| mm-HR4X-Ager-350 | CGGTAGTTGGACTTGACCTCCTTATACGTCGAGTTGAACATAAGTGCG |
| mm-HL4X-Ager-1211 | TTAGTAGGCGAACTTACGTCGTTATGTCCGCTTCCTCTGACTGATT |
| mm-HR4X-Ager-1211 | GGCACCATTCTCTGGCATCTTATACGTCGAGTTGAACATAAGTGCG |
| mm-HL4X-Ager-976 | TTAGTAGGCGAACTTACGTCGTTATGTGACAGGAGGGCTTTCCTG |
| mm-HR4X-Ager-976 | GGTTTCTGTGACCCTGATGCTTATACGTCGAGTTGAACATAAGTGCG |
| mm-HL3X-Ftl1-808 | TATTCGTTCGAACTTACGTCGTTATGTCCTAGTCGTGCTTGAGAGTGA |
| mm-HR3X-Ftl1-808 | CCTTGGAAGGTACAGAGGCCTTATACGTCGAGTTGACCGACGTATTG |
| mm-HL3X-Ftl1-515 | TATTCGTTCGAACTTACGTCGTTATGTTGAGATGGCTTCTGCACAT |
| mm-HR3X-Ftl1-515 | TGGGTTTTACCCCATTCATCTTATACGTCGAGTTGACCGACGTATTG |
| mm-HL3X-Ftl1-152 | TATTCGTTCGAACTTACGTCGTTATGTTGTAAAGGCGGCTGG AAG |
| mm-HR3X-Ftl1-152 | AGGCTGCGACTGGAGAGAC:TTATACGTCGAGTTGACCGACGTATTG |
| mm-HL3X-Ftl1-204 | TATTCGTTCGAACTTACGTCGTTATG:TAGGAGCTGAAGGCAG CG |
| mm-HR3X-Ftl1-204 | GAC GGT GCA GAC TGG TCC:TTATACGTCGAGTTGACCGACGTATTG |
| mm-HL3X-Ftl1-152 | TATTCGTTCGAACTTACGTCGTTATG:T TGT AAA GGC GGC TGG AAG |
| mm-HR3X-Ftl1-152 | AGGCTGCGACTGGAGAGA C:TTATACGTCGAGTTGACCGACGTATTG |
| mm-HL3X-Ftl1-204 | TATTCGTTCGAACTTACGTCGTTATG:T AGG AGC TGA AGG CAG CG |
| mm-HR3X-Ftl1-204 | GAC GGT GCA GAC TGG TCC:TTATACGTCGAGTTGACCGACGTATTG |
| mm-HL3X-Ftl1-808 | TATTCGTTCGAACTTACGTCGTTATG:TCCTAGTCGTGCTTGAGAGTGA |
| mm-HR3X-Ftl1-808 | CCTTGGAAGGTACAGAGGCC:TTATACGTCGAGTTGACCGACGTATTG |
| mm-HL3X-Ftl1-515 | TATTCGTTCGAACTTACGTCGTTATG:TTGAGATGGCTTCTGCACAT |
| mm-HR3X-Ftl1-515 | TGGGTTTTACCCCATTCATC:TTATACGTCGAGTTGACCGACGTATTG |
| mm-HL3X-Ftl1-391 | TATTCGTTCGAACTTACGTCGTTATGTCCAGAGCCACGTCATCC |
| mm-HR3X-Ftl1-391 | AAGAAGTGGCCTACGCCCTTATACGTCGAGTTGACCGACGTATTG |

| mm-HL5X-Scgb1a1-229 | TAGCGCTAACAACTTACGTCGTTATGTCTTCAGCTGGGTGCCC |
| --- | --- |
| mm-HR5X-Scgb1a1-229 | GGGAGGGTATCCACCAGTCTTATACGTCGAGTTGAACGTCGTA  ACA |
| mm-HL5X-Scgb1a1-367 | TAGCGCTAACAACTTACGTCGTTATGTCTGAAATCCAGTGAG  CTTCAG |
| mm-HR5X-Scgb1a1-367 | TGACAAGGCTTTAGCAGTAGAATATCTTATACGTCGAGTTG  AACGTCGTAACA |

| mm-HL2X-Sftpc-783 | TCGTACGTCTAACTTACGTCGTTATGTTTATTCTTTTGTGATAGGATCCC |
| --- | --- |
| mm-HR2X-Sftpc-783 | TTGTTTTCCAATCAGGCTGCTT ATA CGTCGAGTTGAAGAACAACCT G |
| mm-HL2X-Sftpc-646 | TCG TAC GTC TAA CTTACGTCGTTATGTGCGGTTTCTACCGACC |
| mm-HR2X-Sftpc-646 | GGTCTTTCCTGTCCCGCTT ATA CGT CGA GTT GAA GAA CAA CCT G |

| mm-HL6X-Actb-610 | TAGGTCAGGAAACTTACGTCGTTATGTCGTAGATGGGCACAGTGTG |
| --- | --- |
| mm-HR6X-Actb-610 | GTG AGGGAGAGCATAGCC CTTATACGTCGAGTTGAATAGCCAGGTT |
| mm-HL6X-Actb-1111 | TAGGTCAGGAAACTTACGTCGTTATGTCAGGAGGAGCAATGA TCTTG |
| mm-HR6X-Actb-1111 | TCCACACAGAGTACTTGCGC:TTATACGTCGAGTTGAATAGCCAGGTT |
| mm-HL6X-Actb-1465 | TAGGTCAGGAAACTTACGTCGTTATGTCCAACCAACTGCTGTCG |
| mm-HR6X-Actb-1465 | AACTTTGGGGGATGTTTGCTTATACGTCGAGTTGAATAGCCAGGTT |
| mm-HL6X-Actb-1771 | TAGGTCAGGAAACTTACGTCGTTATGTGGGCCATTCAGAAATTAA AA |
| mm-HR6X-Actb-1771 | AAAAGGGAGGCCTCAGACCTTATACGTCGAGTTGAATAGCCAGGTT |

| mm-HL11X-Gapdh-360 | TGTTCGTGTTAACTTACGTCGTTATGACTGGAACATGTAGACCATG |
| --- | --- |
| mm-HR11X-Gapdh-360 | TTTGCCGTGAGTGGAGTCATTTATACGTCGAGTTGAATCTTGATTCGGT |
| mm-HL11X-Gapdh-632 | TGTTCGTGTTAACTTACGTCGTTATGATTTCTCGTGGTTCACACCC |
| mm-HR11X-Gapdh-632 | AATCTTGAGTGAGTTGTCATTTATACGTCGAGTTGAATCTTGATTCGGT |
| mm-HL11X-Gapdh-923 | TGTTCGTGTTAACTTACGTCGTTATGAGGAACACGGAAGGCCATGC |
| mm-HR11X-Gapdh-923 | ACGACGGACACATTGGGGGTTTATACGTCGAGTTGAATCTTGATTCGGT |
| mm-HL11X-Gapdh-1155 | TGTTCGTGTTAACTTACGTCGTTATGACCAGGAAATGAGCTTGACA |
| mm-HR11X-Gapdh-1155 | GTAGCCGTATTCATTGTCATTTATACGTCGAGTTGAATCTTGATTCGGT |
| mm-HL11X-Gapdh-1385 | TGTTCGTGTTAACTTACGTCGTTATGATTGATGGTATTCAAGAGAG |
| mm-HR11X-Gapdh-1385 | TTGTGGGTGCAGCGAACTTTTTATACGTCGAGTTGAATCTTGATTCGGT |

| mm-HL7X-Xist-1447 | GTGTAATTGTCACTTACGTCGTTATGTTAGACACATTCAAGAGCAT |
| --- | --- |
| mm-HR7X-Xist-1447 | TCACATGACTTCCGCCATCTTATACGTCGAGTTGATATTCCTCAGG |
| mm-HL7X-Xist-2130 | GTGTAATTGTCACTTACGTCGTTATGTTGAGATCAGTGCTGGCTAA |
| mm-HR7X-Xist-2130 | CAGTAGGCTTAGAGAACCGCTTATACGTCGAGTTGATATTCCTCAGG |
| mm-HL7X-Xist-8362 | GTGTAATTGTCACTTACGTCGTTATGTAAGCTTCCTTCTCAGGAAG |
| mm-HR7X-Xist-8362 | AAGAACGGAAAGAAAAGTGCTTATACGTCGAGTTGATATTCCTCAGG |
| mm-HL7X-Xist-10145 | GTGTAATTGTCACTTACGTCGTTATGTTGATCACGCTGAAGACCCA |
| mm-HR7X-Xist-10145 | CTTTTGTGTGTTCACATTGCTTATACGTCGAGTTGATATTCCTCAGG |
| mm-HL7X-Xist-16942 | GTGTAATTGTCACTTACGTCGTTATGTTTCGTTCCTCCAGTTTTCT |
| mm-HR7X-Xist-16942 | CAAGTTCTTTCAATTTGGTCTTATACGTCGAGTTGATATTCCTCAGG |

| mm-HL12X-Lyz2-920 | TCAAGTATGGTACTTACGTCGTTATGTCCTGTGCCCTCAGAAACCT |
| --- | --- |
| mm-HR12X-Lyz2-920 | TCAGTTCTCATCCACTGAGACTTATACGTCGAGTTGAGATGTATCCTG |
| mm-HL12X-Lyz2-791 | TCAAGTATGGTACTTACGTCGTTATGTTGTATTTTTATTCACACAG |
| mm-HR12X-Lyz2-791 | TGGTCTCCTATAAGCAGTTCTTATACGTCGAGTTGAGATGTATCCTG |
| mm-HL12X-Lyz2-607 | TCAAGTATGGTACTTACGTCGTTATGTGAAGCTTGTGAGGAGGAAG |
| mm-HR12X-Lyz2-607 | TTGCACAACACAGCATCAGCTTATACGTCGAGTTGAGATGTATCCTG |
| mm-HL12X-Lyz2-422 | TCAAGTATGGTACTTACGTCGTTATGTCGGTTTTGACAGTGTGCTC |
| mm-HR12X-Lyz2-422 | CGAATATACTGGGACAGATCTTATACGTCGAGTTGAGATGTATCCTG |
| mm-HL12X-Lyz2-246 | TCAAGTATGGTACTTACGTCGTTATGTATTGATCTGAAATATCCCA |
| mm-HR12X-Lyz2-246 | ATCATTACACCAGTATCGGCTTATACGTCGAGTTGAGATGTATCCTG |
| mm-HL12X-Lyz2-67 | TCAAGTATGGTACTTACGTCGTTATGTTGGCCTGAGCAGTGACAGA |
| mm-HR12X-Lyz2-67 | ACTCACAACGTTCATAGACCTTATACGTCGAGTTGAGATGTATCCTG |

Blocking oligonucleotides - Scgb1a1

| TRN10+HLR+10-Scgb1a1-248 | GGTCTCTTGTGGGAGGGTATCCACCAGTCTCTT  CAGCTGGGTGCCCGCATTTTGCA |
| --- | --- |
| Control Scrambled oligo | TATCGTGTAATCAGTCTCCCAAAGACTCGGACAACATAACGACGTAAGTTAGACGTACGA |

Short imager oligo

| ImO-Y1-A488-2 | /5Alex488N/TGAGCTAGAAT |
| --- | --- |

Mouse Axin2 - PLISH probes

| mm-HL3X-Axin2-2956 | TATTCGTTCGAACTTACGTCGTTATG:ATTTCGTGGCTGTTGCGTA |
| --- | --- |
| mm-HR3X-Axin2-2956 | TTTCATTTTCCTTCAGAAT:TTATACGTCGAGTTGACCGACGTATTG |
| mm-HL3X-Axin2-2412 | TATTCGTTCGAACTTACGTCGTTATG:AGCTGTGCCAAAGTGTTGG |
| mm-HR3X-Axin2-2412 | CCTGCGGCAGGCTTCCTCT:TTATACGTCGAGTTGACCGACGTATTG |
| mm-HL3X-Axin2-1878 | TATTCGTTCGAACTTACGTCGTTATG:AGTGGTGGTGAACGTGCT |
| mm-HR3X-Axin2-1878 | ACAGCGTGGTGGTGGATGT:TTATACGTCGAGTTGACCGACGTATTG |
| mm-HL3X-Axin2-347 | TATTCGTTCGAACTTACGTCGTTATG:AACACGGCGCTACTCATGGT |
| mm-HR3X-Axin2-347 | GATCTGGAAGGAGAGTCACT:TTATACGTCGAGTTGACCGACGTATTG |

Mouse Axin2 - HCR probes

| HCR1-H1-A488 | GAAGCGAATATGGTGAGAGTTGGAGGTAGGT  TGAGGCACATTTACAGACCTCAACCTACCTCCAACTCTCAC |
| --- | --- |
| HCR1-H2-A488 | CCTCAACCTACCTCCAACTCTCACCATATTCGCTTCGTGAG  AGTTGGAGGTAGGTTGAGGTCTGTAAATGTG |
| HCR1-Axin2-621-A488 | CCTCAACCTACCTCCAACTCTCACCATATTCGCTTCT  AAAACGTATCCACACATTTCTCCCTCTCCAGGAAAGTCCGGA  AGAGGTATGCACATTTTCACATTTACAGACCTCAA  CCTACCTCCAACTCTCAC |
| HCR2-H1-A647 | GGCGGTTTACTGGATGATTGATGAGGATTTACGAGGA  GCTCAGTCCATCCTCGTAAATCCTCATCAATCATC |
| HCR2-H2-A647 | /5Cy5/CCTCGTAAATCCTCATCAATCATCCAGTAAACC  GCCGATGATTGATGAGGATTTACGAGGATGGACTGAGCT |
| HCR2-Axin2-1159-A647 | CCTCGTAAATCCTCATCAATCATCCAGTAAACCGCCA  AAAAAACCTACGTGATAAGGATTGACTGGGTCGCTTC  TCTTGAAGGACCTGAATAAAAAAGCTCAGTCCATCCT  CGTAAATCCTCATCAATCATCC |

Human PLISH probes

| HL4X-MLLT10-4613 | TTAGTAGGCGAACTTACGTCGTTATGTGTCACATCAAAAGCGTGG |
| --- | --- |
| HR4X-MLLT10-4613 | GGGGAATCACTCTGCGGTACTTATACGTCGAGTTGAACATAAGTGCG |
| HL4X-MLLT10-4532 | TTAGTAGGCGAACTTACGTCGTTATGTTTTAATGGTCAGTAATGGGAAGTG |
| HR4X-MLLT10-4532 | TTTCAGGAACTCTAGTGGTGAGCTTATACGTCGAGTTGAACATAAGTGCG |
| HL5X-MLLT10-4613 | TAGCGCTAACAACTTACGTCGTTATGTGTCACATCAAAAGCGTGG |
| HL5X-MLLT10-4418 | TAGCGCTAACAACTTACGTCGTTATGTGAGCTATCTACAGTGGCACAGA |
| HL5X-MLLT10-4277 | TAGCGCTAACAACTTACGTCGTTATGTTGATCAAGGGCAAACCC |
| HL5X-MLLT10-3629 | TAGCGCTAACAACTTACGTCGTTATGTTACAAGAATGTCCTTGCAGCA |
| HR5X-MLLT10-4613 | GGGGAATCACTCTGCGGTACTTATACGTCGAGTTGAACGTCGTAACA |
| HR5X-MLLT10-4418 | AAATACCCAGGTAATAGTTTTGCCTTATACGTCGAGTTGAACGTCGTAACA |
| HR5X-MLLT10-4277 | GCAGCACAATGGAGGCACTTATACGTCGAGTTGAACGTCGTAACA |
| HR5X-MLLT10-3629 | GCAACAAGAAAACTAATCAAAGCCTTATACGTCGAGTTGAACGTCGTAACA |
| HL5X-MLLT10-4532 | TAGCGCTAACAACTTACGTCGTTATGTTTTAATGGTCAGTAATGGGAAGTG |
| HL5X-MLLT10-4338 | TAGCGCTAACAACTTACGTCGTTATGTGCTCGGAGGACTGACTGT |
| HL5X-MLLT10-4177 | TAGCGCTAACAACTTACGTCGTTATGTCACTTAACTCAAGATTTCTTCAAAA |
| HL5X-MLLT10-3579 | TAGCGCTAACAACTTACGTCGTTATGTTGTTGCGTTTCTTCATAGCTG |
| HL5X-MLLT10-2221 | TAGCGCTAACAACTTACGTCGTTATGTCCTACTTGTTGCAAATGCC |
| HL5X-MLLT10-1312 | TAGCGCTAACAACTTACGTCGTTATGTGAAGATTTCTTCCCTTTGCC |
| HR5X-MLLT10-1312 | CTTTGACCTGAGCTGTGAGCTTATACGTCGAGTTGAACGTCGTAACA |
| HR5X-MLLT10-4532 | TTTCAGGAACTCTAGTGGTGAGCTTATACGTCGAGTTGAACGTCGTAACA |
| HR5X-MLLT10-4338 | GTGACGGGATGATTCCTGCTTATACGTCGAGTTGAACGTCGTAACA |
| HR5X-MLLT10-4177 | CCCCAGCAGCCTCAGAACTTATACGTCGAGTTGAACGTCGTAACA |
| HR5X-MLLT10-3579 | CCTTTGTTGTGCATTGAGTTTCTTATACGTCGAGTTGAACGTCGTAACA |
| HR5X-MLLT10-2221 | AGCTGAGGGAGAGAGCGCTTATACGTCGAGTTGAACGTCGTAACA |
| HL2X-MLLT10-4613 | TCGTACGTCTAACTTACGTCGTTATGTGTCACATCAAAAGCGTGG |
| HL2X-MLLT10-4418 | TCGTACGTCTAACTTACGTCGTTATGTGAGCTATCTACAGTGGCACAGA |
| HL2X-MLLT10-4277 | TCGTACGTCTAACTTACGTCGTTATGTTGATCAAGGGCAAACCC |
| HL2X-MLLT10-3629 | TCGTACGTCTAACTTACGTCGTTATGTTACAAGAATGTCCTTGCAGCA |
| HL2X-MLLT10-2559 | TCGTACGTCTAACTTACGTCGTTATGTCCAAAAGCTGTTCTATGCTG |
| HL2X-MLLT10-1475 | TCGTACGTCTAACTTACGTCGTTATGTCAGGAAATCCTGGGGAGAC |
| HR2X-MLLT10-4613 | GGGGAATCACTCTGCGGTACTTATACGTCGAGTTGAAGAACAACCTG |
| HR2X-MLLT10-4418 | AAATACCCAGGTAATAGTTTTGCCTTATACGTCGAGTTGAAGAACAACCTG |
| HR2X-MLLT10-4277 | GCAGCACAATGGAGGCACTTATACGTCGAGTTGAAGAACAACCTG |
| HR2X-MLLT10-3629 | GCAACAAGAAAACTAATCAAAGCCTTATACGTCGAGTTGAAGAACAACCTG |
| HR2X-MLLT10-2559 | GTCCTTCACTCCACTGCCTCTTATACGTCGAGTTGAAGAACAACCTG |
| HR2X-MLLT10-1475 | CGCAGATCTGAGTCTGTAAAGCTTATACGTCGAGTTGAAGAACAACCTG |
| HL6X-ETV5-1840 | TAGGTCAGGAAACTTACGTCGTTATGTTGTTTGCCTGAATGGGAA |
| HR6X-ETV5-1840 | CACAAACAAAACCACTGCCCTTATACGTCGAGTTGAATAGCCAGGTT |
| HL6X-ETV5-1911 | TAGGTCAGGAAACTTACGTCGTTATGTTGGGTTCTCCAGATAATACTCAA |
| HR6X-ETV5-1911 | GTGCCAATCCAGAGACAGCTTATACGTCGAGTTGAATAGCCAGGTT |
| HL2X-ETV5-1424 | TCGTACGTCTAACTTACGTCGTTATGTCGACCTGTCCAGGCAAT |
| HR2X-ETV5-1424 | ATCAGCTTGAACTCCATGCCTTATACGTCGAGTTGAAGAACAACCTG |
| HL2X-ETV5-1709 | TCGTACGTCTAACTTACGTCGTTATGTTCAAAGTGGGTCAGCGG |
| HR2X-ETV5-1709 | GAGGTAAGCGGGGCTGTCTTATACGTCGAGTTGAAGAACAACCTG |
| HL2X-ETV5-3344 | TCGTACGTCTAACTTACGTCGTTATGTGCCTCCTTCACATACGAGG |
| HR2X-ETV5-3344 | CACACTCACGGAGTCAGCACTTATACGTCGAGTTGAAGAACAACCTG |
| HL2X-ETV5-3453 | TCGTACGTCTAACTTACGTCGTTATGTCTTCACAAATGTGCTGTTGG |
| HR2X-ETV5-3453 | CCCTTATCCCTGCGAACCTTATACGTCGAGTTGAAGAACAACCTG |
| HL2X-ETV5-1821 | TCGTACGTCTAACTTACGTCGTTATGTTGTTTGCCTGAATGGGAA |
| HR2X-ETV5-1840 | CACAAACAAAACCACTGCCCTTATACGTCGAGTTGAAGAACAACCTG |
| HL5X-ETV5-3453 | TAGCGCTAACAACTTACGTCGTTATGTCTTCACAAATGTGCTGTTGG |
| HR5X-ETV5-3453 | CCCTTATCCCTGCGAACCTTATACGTCGAGTTGAACGTCGTAACA |
| HL5X-ETV5-222 | TAGCGCTAACAACTTACGTCGTTATGTTTGAGAGGTTTCAGCATTGAG |
| HR5X-ETV5-222 | GCTTTCAGCGTCTCTAATACCACTTATACGTCGAGTTGAACGTCGTAACA |
| HL5X-ETV5-1132 | TAGCGCTAACAACTTACGTCGTTATGTCCTGCTTGATTCCCATTG |
| HR5X-ETV5-1132 | ACGCAGTAATCCCGAGGCTTATACGTCGAGTTGAACGTCGTAACA |
| HL5X-ETV5-1991 | TAGCGCTAACAACTTACGTCGTTATGTTTTGGTGGTTTTCTGCCC |
| HR5X-ETV5-1991 | AAGAGTTGAGGCACTGGCCTTATACGTCGAGTTGAACGTCGTAACA |
| HL5X-ETV5-2091 | TAGCGCTAACAACTTACGTCGTTATGTGAACAAGATATTGCTTTGCATG |
| HR5X-ETV5-2091 | CCGTTTTGCGGGTACTAACCTTATACGTCGAGTTGAACGTCGTAACA |
| HL5X-ETV5-2423 | TAGCGCTAACAACTTACGTCGTTATGTACTTGGCGACCACATGG |
| HR5X-ETV5-2423 | AGAGAAAGGGGGCTTGCTCTTATACGTCGAGTTGAACGTCGTAACA |
| HL5X-ETV5-2508 | TAGCGCTAACAACTTACGTCGTTATGTGTCAAAAGTGTTAATCGCCC |
| HR5X-ETV5-2508 | CAAATCAGGGCAAAACAAAATACTTATACGTCGAGTTGAACGTCGTAACA |
| HL5X-ETV5-2672 | TAGCGCTAACAACTTACGTCGTTATGTGGCTAAAGGACACAGTGCA |
| HR5X-ETV5-2672 | CATAAGTTGGCACGGCCCTTATACGTCGAGTTGAACGTCGTAACA |
| HL5X-ETV5-2837 | TAGCGCTAACAACTTACGTCGTTATGTGCCCTGCACACTTCAAA |
| HR5X-ETV5-2837 | GAATCTAAGCTTGTGTCCAGGCTTATACGTCGAGTTGAACGTCGTAACA |
| HL5X-ETV5-1887 | TAGCGCTAACAACTTACGTCGTTATGTTGGGTTCTCCAGATAATACTCAA |
| HR5X-ETV5-1911 | GTGCCAATCCAGAGACAGCTTATACGTCGAGTTGAACGTCGTAACA |
| HL3X-CASP9-1542 | TATTCGTTCGAACTTACGTCGTTATGTCTTCCTCCACTGTTCAGCA |
| HR3X-CASP9-1542 | ACGGCATTCATCTGTCCCTTATACGTCGAGTTGACCGACGTATTG |
| HL3X-CASP9-972 | TATTCGTTCGAACTTACGTCGTTATGTTTCTGCTCCCCACCACAG |
| HR3X-CASP9-972 | CCACCTCAAACCCATGGTCTTATACGTCGAGTTGACCGACGTATTG |
| HL5X-CASP9-1850 | TAGCGCTAACAACTTACGTCGTTATGTACAGACCAGTGGTTGTCAGG |
| HR5X-CASP9-1850 | TTGTGTGTAGCCAAAAATCCCTTATACGTCGAGTTGAACGTCGTAACA |
| HL5X-CASP9-1789 | TAGCGCTAACAACTTACGTCGTTATGTGAGAACCTTTTTGTTTGGCA |
| HR5X-CASP9-1789 | CAGGACATATTTGGCAACACCTTATACGTCGAGTTGAACGTCGTAACA |
| HL5X-CASP9-1695 | TAGCGCTAACAACTTACGTCGTTATGTCACAGCAAAGGGTGACCT |
| HR5X-CASP9-1695 | CCACAATGTACAGGACAGCCTTATACGTCGAGTTGAACGTCGTAACA |
| HL5X-CASP9-1292 | TAGCGCTAACAACTTACGTCGTTATGTGTTTATAAATCCCTTTCACCGA |
| HR5X-CASP9-1292 | GAAATTAAAGCAACCAGGCATCTTATACGTCGAGTTGAACGTCGTAACA |
| HL5X-CASP9-1211 | TAGCGCTAACAACTTACGTCGTTATGTCAAAGATGTCGTCCAGGGT |
| HR5X-CASP9-1211 | TTCAGAGTGAGCCCACTGCTTATACGTCGAGTTGAACGTCGTAACA |
| HL5X-CASP9-1096 | TAGCGCTAACAACTTACGTCGTTATGTAGATATGGCGTCCAGCTGG |
| HR5X-CASP9-1096 | CACTGGGTGTGGGCAAACTTATACGTCGAGTTGAACGTCGTAACA |
| HL5X-CASP9-881 | TAGCGCTAACAACTTACGTCGTTATGTTCTCGACCGACACAGGG |
| HR5X-CASP9-881 | CCCATTGAAGATGTTCACAATCTTATACGTCGAGTTGAACGTCGTAACA |
| HL5X-CASP9-701 | TAGCGCTAACAACTTACGTCGTTATGTCCACCATGAAATGCAGC |
| HR5X-CASP9-701 | AGTCAGGTCGCCCTTCACCTTATACGTCGAGTTGAACGTCGTAACA |
| HL5X-CASP9-341 | TAGCGCTAACAACTTACGTCGTTATGTGGCCTGTGTCCTCTAAGCA |
| HR5X-CASP9-341 | AAACGAAGCCAGCATGTCCTTATACGTCGAGTTGAACGTCGTAACA |
| HL5X-CASP9-284 | TAGCGCTAACAACTTACGTCGTTATGTCCAGATCTATGATCAGCTGC |
| HR5X-CASP9-284 | AGCCTGACTCCCTCGAGTCTTATACGTCGAGTTGAACGTCGTAACA |
| HL2X-CASP9-1602 | TCGTACGTCTAACTTACGTCGTTATGTAGCCCTGGACCAGCCAC |
| HR2X-CASP9-1602 | GGATCATGGGACACAAGTCACTTATACGTCGAGTTGAAGAACAACCTG |
| HL2X-CASP9-1542 | TCGTACGTCTAACTTACGTCGTTATGTCTTCCTCCACTGTTCAGCA |
| HR2X-CASP9-1542 | ACGGCATTCATCTGTCCCTTATACGTCGAGTTGAAGAACAACCTG |
| HL2X-CASP9-1499 | TCGTACGTCTAACTTACGTCGTTATGTAAGAGCCTGTCTGTCACTGG |
| HR2X-CASP9-1499 | GTCGTCAATCTGGAAGCTGCTTATACGTCGAGTTGAAGAACAACCTG |
| HL2X-CASP9-1417 | TCGTACGTCTAACTTACGTCGTTATGTCAGCCTCTTTCAGGCCTG |
| HR2X-CASP9-1417 | TGCAGGAAAGTCCAGGCCTTATACGTCGAGTTGAAGAACAACCTG |
| HL2X-CASP9-1024 | TCGTACGTCTAACTTACGTCGTTATGTGCCAGGGGACTCGTCTT |
| HR2X-CASP9-1024 | CATCTGGCTCGGGGTTACTTATACGTCGAGTTGAAGAACAACCTG |
| HL2X-CASP9-972 | TCGTACGTCTAACTTACGTCGTTATGTTTCTGCTCCCCACCACAG |
| HR2X-CASP9-972 | CCACCTCAAACCCATGGTCTTATACGTCGAGTTGAAGAACAACCTG |
| HL2X-CASP9-543 | TCGTACGTCTAACTTACGTCGTTATGTGCATTTCCCCTCAAACTCT |
| HR2X-CASP9-543 | GCTCAGGATGTAAGCCAAATCTTATACGTCGAGTTGAAGAACAACCTG |
| HL2X-CASP9-475 | TCGTACGTCTAACTTACGTCGTTATGTGGGTGTTTCCGGTCTGAG |
| HR2X-CASP9-475 | CAGAACCAATGTCCACTGGTCTTATACGTCGAGTTGAAGAACAACCTG |
| HL2X-CASP9-395 | TCGTACGTCTAACTTACGTCGTTATGTTCGACAACTTTGCTGCTTG |
| HR2X-CASP9-395 | GGTAAGGTTTTCTAGGGTTGGCTTATACGTCGAGTTGAAGAACAACCTG |
| HL2X-CASP9-82 | TCGTACGTCTAACTTACGTCGTTATGTAAGACTCCAGGCCGCCT |
| HR2X-CASP9-82 | CCATGGCGAGTAGCCAACTTATACGTCGAGTTGAAGAACAACCTG |
| HL2X-GAPDH-1085 | TCGTACGTCTAACTTACGTCGTTATGTACTTCTCATGGTTCACACCCA |
| HR2X-GAPDH-1107 | GATGATCTTGAGGCTGTTGTCATTATACGTCGAGTTGAAGAACAACCTG |
| HL2X-GAPDH-1329 | TCGTACGTCTAACTTACGTCGTTATGGATGACCTTGCCCACAGCCT |
| HR2X-GAPDH-1349 | AGCTTCCCGTTCAGCTCAGGTTATACGTCGAGTTGAAGAACAACCTG |
| HL2X-GAPDH-1757 | TCGTACGTCTAACTTACGTCGTTATGGACTGAGTGTGGCAGGGACT |
| HR2X-GAPDH-1777 | GGAGATTCAGTGTGGTGGGGTTATACGTCGAGTTGAAGAACAACCTG |
| HL2X-GAPDH-1334 | TCGTACGTCTAACTTACGTCGTTATGTCAGGGATGACCTTGCCC |
| HR2X-GAPDH-1353 | AGTGAGCTTCCCGTTCAGCTTATACGTCGAGTTGAAGAACAACCTG |
| HL5X-GAPDH-1755 | TAGCGCTAACAACTTACGTCGTTATGTGAGTGTGGCAGGGACTCC |
| HL5X-GAPDH-1715 | TAGCGCTAACAACTTACGTCGTTATGTTCCTCTTGTGCTCTTGCTG |
| HL5X-GAPDH-1634 | TAGCGCTAACAACTTACGTCGTTATGTGTTGCTGTAGCCAAATTCG |
| HL5X-GAPDH-1500 | TAGCGCTAACAACTTACGTCGTTATGTCAGTGTAGCCCAGGATGC |
| HL5X-GAPDH-1390 | TAGCGCTAACAACTTACGTCGTTATGTGACACGTTGGCAGTGGG |
| HL5X-GAPDH-1311 | TAGCGCTAACAACTTACGTCGTTATGTTGGCAGCGCCAGTAGAG |
| HL5X-GAPDH-1224 | TAGCGCTAACAACTTACGTCGTTATGTTCTGGGTGGCAGTGATG |
| HL5X-GAPDH-984 | TAGCGCTAACAACTTACGTCGTTATGTCCATGGTGGTGAAGACG |
| HL5X-GAPDH-903 | TAGCGCTAACAACTTACGTCGTTATGTCCTGGAAGATGGTGATGG |
| HR5X-GAPDH-1755 | ATTCAGTGTGGTGGGGGACTTATACGTCGAGTTGAACGTCGTAACA |
| HR5X-GAPDH-1715 | CAGCAGTGAGGGTCTCTCTCTTATACGTCGAGTTGAACGTCGTAACA |
| HR5X-GAPDH-1634 | CCATGAGGTCCACCACCCTTATACGTCGAGTTGAACGTCGTAACA |
| HR5X-GAPDH-1500 | AGAGGAGACCACCTGGTGCTTATACGTCGAGTTGAACGTCGTAACA |
| HR5X-GAPDH-1390 | GCAGGTCAGGTCCACCACTTATACGTCGAGTTGAACGTCGTAACA |
| HR5X-GAPDH-1311 | ATGACCTTGCCCACAGCCTTATACGTCGAGTTGAACGTCGTAACA |
| HR5X-GAPDH-1224 | GAGGGGCCATCCACAGTCTTATACGTCGAGTTGAACGTCGTAACA |
| HR5X-GAPDH-984 | AAATGAGCCCCAGCCTTCTTATACGTCGAGTTGAACGTCGTAACA |
| HR5X-GAPDH-903 | GATTTTGGAGGGATCTCGCTTATACGTCGAGTTGAACGTCGTAACA |
| HL5X1-GAPDH-1757 | TAGCGCTAACATTAAGACGCTTGACTGAGTGTGGCAGGGACT |
| HR5X1-GAPDH-1777 | GGAGATTCAGTGTGGTGGGGAAATATGACAGAACACGTCGTAACA |
| HL5X-GAPDH-1757 | TAGCGCTAACAACTTACGTCGTTATGGACTGAGTGTGGCAGGGACT |
| HR5X-GAPDH-1777 | GGAGATTCAGTGTGGTGGGGTTATACGTCGAGTTGAACGTCGTAACA |
| HL4X-GAPDH-1084 | TTAGTAGGCGAACTTACGTCGTTATGACTTCTCATGGTTCACACCCA |
| HR4X-GAPDH-1107 | GATGATCTTGAGGCTGTTGTCATTTATACGTCGAGTTGAACATAAGTGCG |
| HL4X-GAPDH-1757 | TTAGTAGGCGAACTTACGTCGTTATGGACTGAGTGTGGCAGGGACT |
| HR4X-GAPDH-1777 | GGAGATTCAGTGTGGTGGGGTTATACGTCGAGTTGAACATAAGTGCG |
| HL3X1-GAPDH-1757 | TATTCGTTCGATTAAGACGCTTGACTGAGTGTGGCAGGGACT |
| HR3X1-GAPDH-1777 | GGAGATTCAGTGTGGTGGGGAAATATGACAGAACCCGACGTATTG |
| HL3X-GAPDH-1757 | TATTCGTTCGAACTTACGTCGTTATGGACTGAGTGTGGCAGGGACT |
| HR3X-GAPDH-1777 | GGAGATTCAGTGTGGTGGGGTTATACGTCGAGTTGACCGACGTATTG |
| HL5X-ACTB-1652 | TAGCGCTAACAACTTACGTCGTTATGTGGGCCATTCTCCTTAGAGA |
| HL5X-ACTB-1490 | TAGCGCTAACAACTTACGTCGTTATGTGCTGTCACCTTCACCGTT |
| HL5X-ACTB-1389 | TAGCGCTAACAACTTACGTCGTTATGTCATCTTGTTTTCTGCGCAA |
| HL5X-ACTB-1272 | TAGCGCTAACAACTTACGTCGTTATGTGCTTGCTGATCCACATCTG |
| HL5X-ACTB-1176 | TAGCGCTAACAACTTACGTCGTTATGTTGATCTTCATTGTGCTGGG |
| HL5X-ACTB-908 | TAGCGCTAACAACTTACGTCGTTATGTCTTCTCCAGGGAGGAGCT |
| HL5X-ACTB-834 | TAGCGCTAACAACTTACGTCGTTATGTCCTTAATGTCACGCACGAT |
| HL5X-ACTB-603 | TAGCGCTAACAACTTACGTCGTTATGTGGATAGCAACGTACATGGC |
| HL5X-ACTB-363 | TAGCGCTAACAACTTACGTCGTTATGTCGTCGCCCACATAGGAAT |
| HR5X-ACTB-1652 | GTGTGGACTTGGGAGAGGACTTATACGTCGAGTTGAACGTCGTAACA |
| HR5X-ACTB-1490 | ATGCTCGCTCCAACCGACTTATACGTCGAGTTGAACGTCGTAACA |
| HR5X-ACTB-1389 | CAAATAAAGCCATGCCAATCTTATACGTCGAGTTGAACGTCGTAACA |
| HR5X-ACTB-1272 | GCCGGACTCGTCATACTCCTTATACGTCGAGTTGAACGTCGTAACA |
| HR5X-ACTB-1176 | GCTCAGGAGGAGCAATGATCTTATACGTCGAGTTGAACGTCGTAACA |
| HR5X-ACTB-908 | CGTCAGGCAGCTCGTAGCTTATACGTCGAGTTGAACGTCGTAACA |
| HR5X-ACTB-834 | GGCGACGTAGCACAGCTTCTTATACGTCGAGTTGAACGTCGTAACA |
| HR5X-ACTB-603 | CGTACAGGGATAGCACAGCCTTATACGTCGAGTTGAACGTCGTAACA |
| HR5X-ACTB-363 | CCTCTCTTGCTCTGGGCCTTATACGTCGAGTTGAACGTCGTAACA |
| HL5X-ACTB-1533 | TAGCGCTAACAACTTACGTCGTTATGTCGGCCACATTGTGAACTT |
| HR5X-ACTB-1533 | CAACAATGTGCAATCAAAGTCCTTATACGTCGAGTTGAACGTCGTAACA |
| HL5X-ACTB-1819 | TAGCGCTAACAACTTACGTCGTTATGACATCTCAAGTTGGGGGACA |
| HR5X-ACTB-1839 | GGGAGACCAAAAGCCTTCATTTATACGTCGAGTTGAACGTCGTAACA |
| HL2X-ACTB-1678 | TCGTACGTCTAACTTACGTCGTTATGTCCCCTGTGTGGACTTGG |
| HR2X-ACTB-1678 | CACGAAAGCAATGCTATCACCTTATACGTCGAGTTGAAGAACAACCTG |
| HL2X-ACTB-463 | TCGTACGTCTAACTTACGTCGTTATGAGAAGGTGTGGTGCCAGATT |
| HR2X-ACTB-481 | CCACACGCAGCTCATTGTTTATACGTCGAGTTGAAGAACAACCTG |
| HL1X-ACTB-463 | GACGCTAATAGACTTACGTCGTTATGAGAAGGTGTGGTGCCAGATT |
| HR1X-ACTB-481 | CCACACGCAGCTCATTGTTTATACGTCGAGTTGATAGACACTCTT |
| HL2X-UBC-2636 | TCGTACGTCTAACTTACGTCGTTATGTTAAAAGGGGAAACTTAGACACCC |
| HR2X-UBC-2659 | GTGCAATGAAATTTGTTGAAACCTTATACGTCGAGTTGAAGAACAACCTG |
| HL2X-UBC-2482 | TCGTACGTCTAACTTACGTCGTTATGTTGGATCTTTGCCTTGACATT |
| HR2X-UBC-2482 | GGAGGGATGCCTTCCTTATCTTATACGTCGAGTTGAAGAACAACCTG |
| HL6X-UBC-2055 | TAGGTCAGGAAACTTACGTCGTTATGTGCTGGTCAGGAGGGATG |
| HR6X-UBC-2055 | TCCCAGCAAAGATCAACCTCTTATACGTCGAGTTGAATAGCCAGGTT |
| HL5X-UBC-2637 | TAGCGCTAACAACTTACGTCGTTATGTTAAAAGGGGAAACTTAGACACCC |
| HL5X-UBC-2580 | TAGCGCTAACAACTTACGTCGTTATGTCTTTCTGGATGTTGTAGTCAGACA |
| HL5X-UBC-2535 | TAGCGCTAACAACTTACGTCGTTATGTGTTTCCCAGCAAAGATCAA |
| HL5X-UBC-2491 | TAGCGCTAACAACTTACGTCGTTATGTTCCTTATCTTGGATCTTTGCC |
| HL5X-UBC-2381 | TAGCGCTAACAACTTACGTCGTTATGTGAGACGGAGCACCAGGT |
| HL5X-UBC-2314 | TAGCGCTAACAACTTACGTCGTTATGTTCCAGCTGTTTCCCAGC |
| HL5X-UBC-2283 | TAGCGCTAACAACTTACGTCGTTATGTGCTGGTCAGGAGGGATG |
| HL5X-UBC-2232 | TAGCGCTAACAACTTACGTCGTTATGTCAATGGTGTCACTCGGCT |
| HL5X-UBC-2153 | TAGCGCTAACAACTTACGTCGTTATGTAAGACGGAGCACCAGGTG |
| HR5X-UBC-2637 | TGCAATGAAATTTGTTGAAACCTTATACGTCGAGTTGAACGTCGTAACA |
| HR5X-UBC-2580 | GGACCAAGTGCAGAGTGGACTTATACGTCGAGTTGAACGTCGTAACA |
| HR5X-UBC-2535 | GTGCGTCCATCTTCCAGCTTATACGTCGAGTTGAACGTCGTAACA |
| HR5X-UBC-2491 | CTGATCAGGAGGGATGCCTTATACGTCGAGTTGAACGTCGTAACA |
| HR5X-UBC-2381 | GAAGATTTGCATCCCACCTCTTATACGTCGAGTTGAACGTCGTAACA |
| HR5X-UBC-2314 | AGACAGGGTGCGTCCATCTTATACGTCGAGTTGAACGTCGTAACA |
| HR5X-UBC-2283 | TCCCAGCAAAGATCAACCTCTTATACGTCGAGTTGAACGTCGTAACA |
| HR5X-UBC-2232 | TGGATCTTTGCCTTGACATTCTTATACGTCGAGTTGAACGTCGTAACA |
| HR5X-UBC-2153 | GAAGATCTGCATCCCACCTCTTATACGTCGAGTTGAACGTCGTAACA |
| HL5X-UBC-2435 | TAGCGCTAACAACTTACGTCGTTATGTCGAGGGTGATGGTCTTACC |
| HR5X-UBC-2453 | GTGTCACTGGGCTCCACCTTATACGTCGAGTTGAACGTCGTAACA |
| HL1X-UBC-2636 | GACGCTAATAGACTTACGTCGTTATGTTAAAAGGGGAAACTTAGACACCC |
| HR1X-UBC-2659 | GTGCAATGAAATTTGTTGAAACCTTATACGTCGAGTTGATAGACACTCTT |
| HL3X-PPIA-1397 | TATTCGTTCGAACTTACGTCGTTATGTCAGGAGGCTGAGGCACTAG |
| HR3X-PPIA-1397 | ACGCCCATTATCCCAGCTACTTATACGTCGAGTTGACCGACGTATTG |
| HL5X-PPIA-2201 | TAGCGCTAACAACTTACGTCGTTATGTCAGGTCTGAGCCACAAGTACA |
| HL5X-PPIA-2124 | TAGCGCTAACAACTTACGTCGTTATGTGAGATGCACAAGTGGTGGT |
| HL5X-PPIA-2028 | TAGCGCTAACAACTTACGTCGTTATGTCTAGGATAGGCAAACTTGATTGC |
| HL5X-PPIA-1982 | TAGCGCTAACAACTTACGTCGTTATGTGTTAAGGTGGGCAGAGAAG |
| HL5X-PPIA-1883 | TAGCGCTAACAACTTACGTCGTTATGTTAGTGGGAAGGGTATCCCTTT |
| HL5X-PPIA-1820 | TAGCGCTAACAACTTACGTCGTTATGTCAAAGCCTCCATAACCAAA |
| HL5X-PPIA-1732 | TAGCGCTAACAACTTACGTCGTTATGTGCAGGGAGACTGACTGTAGC |
| HR5X-PPIA-2201 | GATGTTTATTTCCACCTTGCACTTATACGTCGAGTTGAACGTCGTAACA |
| HR5X-PPIA-2124 | CGTAACCAGACAACACACAAGACTTATACGTCGAGTTGAACGTCGTAACA |
| HR5X-PPIA-2028 | AATGATCAAATCCGCCACCTTATACGTCGAGTTGAACGTCGTAACA |
| HR5X-PPIA-1982 | TGGGTTAAGAACCCTAGAGGTCTTATACGTCGAGTTGAACGTCGTAACA |
| HR5X-PPIA-1883 | AATTTCCTGAGAAACCAAGTCCTTATACGTCGAGTTGAACGTCGTAACA |
| HR5X-PPIA-1820 | TTGGTCAGGTTTGCAAAACCTTATACGTCGAGTTGAACGTCGTAACA |
| HR5X-PPIA-1732 | TAGTCTGCGCCTTAACCCTCTTATACGTCGAGTTGAACGTCGTAACA |
| HL5X-PPIA-378 | TAGCGCTAACAACTTACGTCGTTATGTGGGAACCATTTGTGTTGG |
| HR5X-PPIA-378 | TGGCAGTGCAGATGAAAAACTTATACGTCGAGTTGAACGTCGTAACA |
| HL5X-PPIA-655 | TAGCGCTAACAACTTACGTCGTTATGTGCAGCGAGAGCACAAAG |
| HR5X-PPIA-655 | ACATGGAACCCAAAGGGAACTTATACGTCGAGTTGAACGTCGTAACA |
| HL5X-PPIA-588 | TAGCGCTAACAACTTACGTCGTTATGTACAGAAGGAATGATCTGGTGG |
| HR5X-PPIA-606 | AGGGGTGCTCTCCTGAGCTTATACGTCGAGTTGAACGTCGTAACA |
| HL2X-PPIA-473 | TCGTACGTCTAACTTACGTCGTTATGTCCATGGCCTCCACAATATT |
| HR2X-PPIA-489 | CCTGGACCCAAAGCGCTTATACGTCGAGTTGAAGAACAACCTG |
| HL1X-PPIA-473 | GACGCTAATAGACTTACGTCGTTATGTCCATGGCCTCCACAATATT |
| HR1X-PPIA-489 | CCTGGACCCAAAGCGCTTATACGTCGAGTTGATAGACACTCTT |
| HL5X-SOX4-5611 | TAGCGCTAACAACTTACGTCGTTATGTTAAAAAGGCAGACGTCCTAGTG |
| HR5X-SOX4-5611 | AAACCCTTAACGGAACTGCCTTATACGTCGAGTTGAACGTCGTAACA |
| HL5X-SOX4-492 | TAGCGCTAACAACTTACGTCGTTATGTCTGGTGCAAAATCTGTTCC |
| HR5X-SOX4-492 | GCATTGGAATAAAGAATCAGCCTTATACGTCGAGTTGAACGTCGTAACA |
| HL5X-SOX4-4150 | TAGCGCTAACAACTTACGTCGTTATGTGCCCCTGTACCACATCAA |
| HR5X-SOX4-4169 | CACTCGCCCTCCACTGACTTATACGTCGAGTTGAACGTCGTAACA |
| HL5X-SOX4-1521 | TAGCGCTAACAACTTACGTCGTTATGTCTCTCCCTCTCTCTCGCTCT |
| HR5X-SOX4-1521 | TCCCAGGCTGGAGAGTCTCTTATACGTCGAGTTGAACGTCGTAACA |
| HL5X-SOX4-2074 | TAGCGCTAACAACTTACGTCGTTATGTGTCTTTGAGCAGCTTCCAG |
| HR5X-SOX4-2074 | AATGAAAGGGATCTTGTCGCTTATACGTCGAGTTGAACGTCGTAACA |
| HL5X-SOX4-3011 | TAGCGCTAACAACTTACGTCGTTATGTCAAAGTTTGAGCTGGGGTT |
| HR5X-SOX4-3011 | CTGCCCAGGGACATGCTCTTATACGTCGAGTTGAACGTCGTAACA |
| HL5X-SOX4-3450 | TAGCGCTAACAACTTACGTCGTTATGTCTTCCTCCTCCGCGTC |
| HR5X-SOX4-3450 | GTCGCCCCTGTCTACCCTTATACGTCGAGTTGAACGTCGTAACA |
| HL5X-SOX4-3809 | TAGCGCTAACAACTTACGTCGTTATGTCAGTCCCGGCGTTCC |
| HR5X-SOX4-3809 | CGCCTGCGTGGAGTCTCTTATACGTCGAGTTGAACGTCGTAACA |
| HL5X-SOX4-4009 | TAGCGCTAACAACTTACGTCGTTATGTTCCAGTTCGTGTCCTCCTC |
| HR5X-SOX4-4009 | AGTTTGACCGTGAACCCCCTTATACGTCGAGTTGAACGTCGTAACA |
| HL5X-SOX4-5145 | TAGCGCTAACAACTTACGTCGTTATGTTTTTACAAGACGGGGGCC |
| HR5X-SOX4-5145 | TTTGGCTAATTCTCCTCCCCTTATACGTCGAGTTGAACGTCGTAACA |
| HL2X-SOX4-4169 | TCGTACGTCTAACTTACGTCGTTATGTGCCCCTGTACCACATCAA |
| HR2X-SOX4-4169 | CACTCGCCCTCCACTGACTTATACGTCGAGTTGAAGAACAACCTG |
| HL2X-SOX4-5611 | TCGTACGTCTAACTTACGTCGTTATGTTAAAAAGGCAGACGTCCTAGTG |
| HR2X-SOX4-5611 | AAACCCTTAACGGAACTGCCTTATACGTCGAGTTGAAGAACAACCTG |
| HL2X-SOX4-3908 | TCGTACGTCTAACTTACGTCGTTATGTCCTCCCCTCAACATCTCCT |
| HR2X-SOX4-3908 | GGTCACACTGGCTGGCCTTATACGTCGAGTTGAAGAACAACCTG |
| HL2X-SOX4-1449 | TCGTACGTCTAACTTACGTCGTTATGTGAGTCTGGAGACCGTGCTA |
| HR2X-SOX4-1469 | CAGTTTGCTGTCTCTCGGCTTATACGTCGAGTTGAAGAACAACCTG |
| HL2X-SOX4-1933 | TCGTACGTCTAACTTACGTCGTTATGTCGGGGTCTTGCACCA |
| HR2X-SOX4-1933 | GTCGCTTGATGTGCCCACTTATACGTCGAGTTGAAGAACAACCTG |
| HL2X-ID2-107 | TCGTACGTCTAACTTACGTCGTTATGTAGCTGCGCTTGGCACC |
| HR2X-ID2-107 | TGCCGCCTGCTGAGCTTATACGTCGAGTTGAAGAACAACCTG |
| HL2X-ID2-224 | TCGTACGTCTAACTTACGTCGTTATGTGTTTTTCCTAACGGACCTCA |
| HR2X-ID2-224 | GCTGTGGTCCGACAGGCTTATACGTCGAGTTGAAGAACAACCTG |
| HL2X-ID2-284 | TCGTACGTCTAACTTACGTCGTTATGTCATCGGGTCGTCCACA |
| HR2X-ID2-284 | TCGTTCATGTTGTATAGCAGGCTTATACGTCGAGTTGAAGAACAACCTG |
| HL2X-ID2-411 | TCGTACGTCTAACTTACGTCGTTATGTGCAGGTCCAAGATGTAGTCG |
| HR2X-ID2-411 | CGAGTCCAGGGCGATCTTATACGTCGAGTTGAAGAACAACCTG |
| HL2X-ID2-854 | TCGTACGTCTAACTTACGTCGTTATGTCCCCATGGTGGGAATAGT |
| HR2X-ID2-873 | CTTGTGATTTTAACGTTTTCGCTTATACGTCGAGTTGAAGAACAACCTG |
| HL5X-ID2-149 | TAGCGCTAACAACTTACGTCGTTATGTGGCTGCCCTGAAGCTC |
| HR5X-ID2-149 | GAGACCGGGAGGGAGCTTATACGTCGAGTTGAACGTCGTAACA |
| HR5X-ID2-190 | ACCTCACGGGACTGAAGGCTTATACGTCGAGTTGAACGTCGTAACA |
| HL5X-ID2-197 | TAGCGCTAACAACTTACGTCGTTATGTGAAGGCTTTCATGCTGACC |
| HL5X-ID2-362 | TAGCGCTAACAACTTACGTCGTTATGTCACCTTCTTGTTCTGGGGG |
| HR5X-ID2-362 | CTGCAGGATTTCCATCTTGCTTATACGTCGAGTTGAACGTCGTAACA |
| HL5X-ID2-468 | TAGCGCTAACAACTTACGTCGTTATGTGCCCGGGTCTCTGGT |
| HR5X-ID2-468 | GTCCTGGACGCCTGGTTCTTATACGTCGAGTTGAACGTCGTAACA |
| HL5X-ID2-552 | TAGCGCTAACAACTTACGTCGTTATGTCAGAAGGGAATTCAGAAGCC |
| HR5X-ID2-552 | GCTTTGCTGTCATTTGACATTAACTTATACGTCGAGTTGAACGTCGTAACA |
| HL5X-ID2-593 | TAGCGCTAACAACTTACGTCGTTATGTTATTCAGCCACACAGTGCTTT |
| HR5X-ID2-593 | AAAAGAAATCATGAACACCGCTTATACGTCGAGTTGAACGTCGTAACA |
| HL5X-ID2-694 | TAGCGCTAACAACTTACGTCGTTATGTCCTTGTGAAATGGTTGAAAAA |
| HR5X-ID2-694 | AAAAGGTCCATTCAACTTGTCCTTATACGTCGAGTTGAACGTCGTAACA |
| HL5X-ID2-951 | TAGCGCTAACAACTTACGTCGTTATGTGGTATTCACGCTCCACCTT |
| HR5X-ID2-951 | AAGTGACTGAATACTGGATCCTTCTTATACGTCGAGTTGAACGTCGTAACA |
| HL5X-ID2-1233 | TAGCGCTAACAACTTACGTCGTTATGTCTGAAATAAAGCAGGCAATCA |
| HR5X-ID2-1255 | AATAAAAATCAAAGCACTGGTCCTTATACGTCGAGTTGAACGTCGTAACA |
| HL5X-ID2-411 | TAGCGCTAACAACTTACGTCGTTATGTGCAGGTCCAAGATGTAGTCG |
| HR5X-ID2-411 | CGAGTCCAGGGCGATCTTATACGTCGAGTTGAACGTCGTAACA |
| HL2X-CTNNB1-1615 | TCGTACGTCTAACTTACGTCGTTATGTCTATACCACCCACTTGGCAG |
| HR2X-CTNNB1-1615 | GGACAGTACGCACAAGAGCCTTATACGTCGAGTTGAAGAACAACCTG |
| HL2X-CTNNB1-2599 | TCGTACGTCTAACTTACGTCGTTATGTGATTGCTGTCACCTGGAGG |
| HR2X-CTNNB1-2599 | CAGTATCAAACCAGGCCAGCTTATACGTCGAGTTGAAGAACAACCTG |
| HL2X-CTNNB1-2732 | TCGTACGTCTAACTTACGTCGTTATGTAAACCACTCCCACCCTACC |
| HR2X-CTNNB1-2732 | TGTGGCAGATTTACAAATAGCCTTATACGTCGAGTTGAAGAACAACCTG |
| HL2X-CTNNB1-3000 | TCGTACGTCTAACTTACGTCGTTATGTCGAGCCCTCTCAGCAAC |
| HR2X-CTNNB1-3000 | GAGATACCAGCCCACCCCTTATACGTCGAGTTGAAGAACAACCTG |
| HL2X-CTNNB1-3021 | TCGTACGTCTAACTTACGTCGTTATGTTGGTTAGTGTGTCAGGCACTT |
| HR2X-CTNNB1-3041 | TGTTCCCATAGGAAACTCAGCTTATACGTCGAGTTGAAGAACAACCTG |
| HL5X-CTNNB1-149 | TAGCGCTAACAACTTACGTCGTTATGTCAGACCTTCCTCCGTCTCC |
| HR5X-CTNNB1-149 | GGGGACTGAAGCTGCTCCTTATACGTCGAGTTGAACGTCGTAACA |
| HL5X-CTNNB1-348 | TAGCGCTAACAACTTACGTCGTTATGTAACAGCCGCTTTTCTGTCTG |
| HR5X-CTNNB1-348 | AGACTGTTGCTGCCAGTGACTTATACGTCGAGTTGAACGTCGTAACA |
| HL5X-CTNNB1-958 | TAGCGCTAACAACTTACGTCGTTATGTCACGATGATGGGAAAGGTT |
| HR5X-CTNNB1-958 | TAAAGATGGCCAGTAAGCCCTTATACGTCGAGTTGAACGTCGTAACA |
| HL5X-CTNNB1-2117 | TAGCGCTAACAACTTACGTCGTTATGTCTTTGGATGTTTTCAATGGG |
| HR5X-CTNNB1-2117 | AGAGGACCCCTGCAGCTACTTATACGTCGAGTTGAACGTCGTAACA |
| HL5X-CTNNB1-2528 | TAGCGCTAACAACTTACGTCGTTATGTGGATAGTCAGCACCAGGGT |
| HR5X-CTNNB1-2528 | ATCTGGCAGCCCATCAACTTATACGTCGAGTTGAACGTCGTAACA |
| HL5X-CTNNB1-3105 | TAGCGCTAACAACTTACGTCGTTATGTCCTCGACCAAAAAGGACC |
| HR5X-CTNNB1-3105 | TCCCAAAATCCATTTGTATTGTTACTTATACGTCGAGTTGAACGTCGTAACA |
| HL5X-CTNNB1-3147 | TAGCGCTAACAACTTACGTCGTTATGTTCACTTCTTGAGTCACTCCCA |
| HR5X-CTNNB1-3147 | TGTGATCCATTCTTGTGCATTCTTATACGTCGAGTTGAACGTCGTAACA |
| HL5X-CTNNB1-3618 | TAGCGCTAACAACTTACGTCGTTATGTGTCCAAAACAAGGTTCCAA |
| HR5X-CTNNB1-3618 | GGGATAAAAGGCAACTGGTAAACTTATACGTCGAGTTGAACGTCGTAACA |
| HL5X-CTNNB1-2580 | TAGCGCTAACAACTTACGTCGTTATGTGATTGCTGTCACCTGGAG |
| HR5X-CTNNB1-2599 | CAGTATCAAACCAGGCCAGCTTATACGTCGAGTTGAACGTCGTAACA |
| HL5X-CTNNB1-3000 | TAGCGCTAACAACTTACGTCGTTATGTCGAGCCCTCTCAGCAAC |
| HR5X-CTNNB1-3000 | GAGATACCAGCCCACCCCTTATACGTCGAGTTGAACGTCGTAACA |
| HL2X-Neg20 | TCGTACGTCTAACTTACGTCGTTATGAACCGGTACGTACGGATTGA |
| HR2X-Neg20 | AACCGGTACGTACGGATTGATTATACGTCGAGTTGAAGAACAACCTG |
| HL5X-Neg20 | TAGCGCTAACAACTTACGTCGTTATGAACCGGTACGTACGGATTGA |
| HR5X-Neg20 | AACCGGTACGTACGGATTGATTATACGTCGAGTTGAACGTCGTAACA |

| HL4X-SCGB1A1a- 91 | TTAGTAGGCGAACTTACGTCGTTATGTGCAGCAGAGAGCCAGTGTG |
| --- | --- |
| HR4X-SCGB1A1a- 112 | TTAGTAGGCGAACTTACGTCGTTATGAACTGGAGGGTGTGTCCATG |
| HL4X-SCGB1A1b-166 | AAGTTCCATGGCAGCCTCATTTATACGTCGAGTTGAACATAAGTGCG |
| HR4X-SCGB1A1b-187 | TTAGTAGGCGAACTTACGTCGTTATGTTAATGATGCTTTCTCTGGG |
| HL4X-SCGB1A1c-278 | GGGCTATTTTTTCCATGAGCTTATACGTCGAGTTGAACATAAGTGCG |
| HR4X-SCGB1A1c-299 | GGGCTATTTTTTCCATGAGCTTATACGTCGAGTTGAACATAAGTGCG |
| HL4X-SCGB1A1d-374 | TTAGTAGGCGAACTTACGTCGTTATGTCAAAGCATGGCAGCGGCAG |
| HR4X-  SCGB1A1d- 395 | CAAGGCTGGTGGGCGTGGACTTATACGTCGAGTTGAACATAAGTGC |
